# Supplementary material for: Gathering the Evidence on Diet and Depression: A Protocol for an Umbrella Review and Updated Meta-Analyses
Source: Methods Protoc. 2023 Aug 31;6(5):78. doi: 10.3390/mps6050078 (PMC10514888; doi:10.3390/mps6050078)
Supplement: Supplementary file 1 [file mps-06-00078-s001.zip › Supplementary file 1. PRISMA-P 2015 checklist.pdf]

Supplementary file 1. PRISMA-P 2015 checklist

This checklist has been adapted for use with systematic review protocol submissions to BioMed Central journals from Table 3 in Moher D et al: Preferred reporting items for systematic review and meta-analysis protocols (PRISMA-P) 2015 statement. *Systematic Reviews* 2015 **4**:1

An Editorial from the Editors-in-Chief of *Systematic Reviews* details why this checklist was adapted - Moher D, Stewart L & Shekelle P: Implementing PRISMA-P: recommendations for prospective authors. *Systematic Reviews* 2016 **5**:15

Section/topic	#	Checklist item	Information reported		Line number(s)
			Yes	No	
ADMINISTRATIVE INFORMATION					
Title: Gathering the evidence on diet and depression: a protocol for an umbrella review and updated meta-analyses					
Identification	1a	Identify the report as a protocol of a systematic review	☒	☐	Lines 2 - 3
Update	1b	If the protocol is for an update of a previous systematic review, identify as such	☒	☐	Lines 2 - 3
Registration	2	If registered, provide the name of the registry (e.g., PROSPERO) and registration number in the Abstract	☒	☐	Line 87<br>Lines 150 - 151
Authors					
Contact	3a	Provide name, institutional affiliation, and e-mail address of all protocol authors; provide physical mailing address of corresponding author	☒	☐	Lines 5 - 48
Contributions	3b	Describe contributions of protocol authors and identify the guarantor of the review	☒	☐	Lines 810 - 816
Amendments	4	If the protocol represents an amendment of a previously completed or published protocol, identify as such and list changes; otherwise, state plan for documenting important protocol amendments	☒	☐	Lines 158 - 159
Support					
Sources	5a	Indicate sources of financial or other support for the review	☒	☐	Lines 802 - 808
Sponsor	5b	Provide name for the review funder and/or sponsor	☒	☐	Lines 803 - 804
Role of sponsor/funder	5c	Describe roles of funder(s), sponsor(s), and/or institution(s), if any, in developing the protocol	☒	☐	Line 804
INTRODUCTION					
Rationale	6	Describe the rationale for the review in the context of what is already known	☒	☐	Lines 95 - 135
Objectives	7	Provide an explicit statement of the question(s) the review will address with reference to participants, interventions, comparators, and outcomes (PICO)	☒	☐	Lines 136 - 147
METHODS					
Eligibility criteria	8	Specify the study characteristics (e.g., PICO, study design, setting, time frame) and report characteristics (e.g., years considered, language, publication status) to be used as criteria for eligibility for the review	☒	☐	Lines 189 - 245

BioMed Central
The Open Access Publisher

Supplementary file 1. PRISMA-P 2015 checklist

Section/topic	#	Checklist item	Information reported		Line number(s)
			Yes	No	
Information sources	9	Describe all intended information sources (e.g., electronic databases, contact with study authors, trial registers, or other grey literature sources) with planned dates of coverage	☒	☐	Lines 165 - 187
Search strategy	10	Present draft of search strategy to be used for at least one electronic database, including planned limits, such that it could be repeated	☒	☐	Table 1 - 2<br>Supp. files 2 – 3
STUDY RECORDS					
Data management	11a	Describe the mechanism(s) that will be used to manage records and data throughout the review	☒	☐	Lines 249 - 274
Selection process	11b	State the process that will be used for selecting studies (e.g., two independent reviewers) through each phase of the review (i.e., screening, eligibility, and inclusion in meta-analysis)	☒	☐	Lines 249 - 258
Data collection process	11c	Describe planned method of extracting data from reports (e.g., piloting forms, done independently, in duplicate), any processes for obtaining and confirming data from investigators	☒	☐	Lines 260 – 274
Data items	12	List and define all variables for which data will be sought (e.g., PICO items, funding sources), any pre-planned data assumptions and simplifications	☒	☐	Table 3 - 4
Outcomes and prioritization	13	List and define all outcomes for which data will be sought, including prioritization of main and additional outcomes, with rationale	☒	☐	Table 3 - 4
Risk of bias in individual studies	14	Describe anticipated methods for assessing risk of bias of individual studies, including whether this will be done at the outcome or study level, or both; state how this information will be used in data synthesis	☒	☐	Line 276 – 332
DATA					
Synthesis	15a	Describe criteria under which study data will be quantitatively synthesized	☒	☐	Line 334 - 409
	15b	If data are appropriate for quantitative synthesis, describe planned summary measures, methods of handling data, and methods of combining data from studies, including any planned exploration of consistency (e.g., I^2, Kendall's tau)	☒	☐	Line 364 - 404
	15c	Describe any proposed additional analyses (e.g., sensitivity or subgroup analyses, meta-regression)	☒	☐	Line 382 - 390
	15d	If quantitative synthesis is not appropriate, describe the type of summary planned	☒	☐	Line 356 - 362
Meta-bias(es)	16	Specify any planned assessment of meta-bias(es) (e.g., publication bias across studies, selective reporting within studies)	☒	☐	Line 406 - 409
Confidence in cumulative evidence	17	Describe how the strength of the body of evidence will be assessed (e.g., GRADE)	☒	☐	Line 411 - 429

BioMed Central
The Open Access Publisher
