# Supplementary material for: Gathering the Evidence on Diet and Depression: A Protocol for an Umbrella Review and Updated Meta-Analyses
Source: Methods Protoc. 2023 Aug 31;6(5):78. doi: 10.3390/mps6050078 (PMC10514888; doi:10.3390/mps6050078)
Supplement: Supplementary file 1 [file mps-06-00078-s001.zip › Supplementary file 2. Search strategy for all databases (UR).pdf]

Supplementary file 2. Search strategy for all databases (umbrella review)

Table S2.1 Full search strategy for Medline (through Ovid; date of last search – 10/06/2020)

Theme or description		Search terms
Diet	1	exp diet/ OR exp nutritive value/ OR exp hunger/ OR appetite regulation/ OR eating/ OR feeding behavior/ OR nutritional physiological phenomena/ OR food/ OR dairy products/ OR dietary carbohydrates/ OR dietary fats/ OR dietary fiber/ OR dietary proteins/ OR dietary supplements/ OR eggs/ OR fast foods/ OR fruit/ OR meals/ OR meat/ OR micronutrients/ OR nuts/ OR seeds/ OR vegetables/
	2	diet*.ti,ab,kw.
	3	nutriti*.ti,ab,kw.
	4	food.ti,ab,kw.
	5	eat*.ti,ab,kw.
	6	energy intake.ti,ab,kw.
	7	(macronutrient* or micronutrient* or nutrient*).ti,ab,kw.
	8	1 OR 2 OR 3 OR 4 OR 5 OR 6 OR 7
Depression	9	exp depressive disorder/ OR depression/ OR mood disorders/
	10	(depression* OR (depressive ADJ3 (condition* OR disorder* OR symptom*))).ti,ab,kw.
	11	9 OR 10
Type of study	12	systematic review/ OR meta-analysis/
	13	(systematic ADJ3 review).ti,ab,kw
	14	meta-analysis OR (meta ADJ2 analysis).ti,ab,kw
	15	12 OR 13 OR 14
Combining search themes	16	8 AND 11 AND 15
Language	17	limit 16 to (english or french)
Search years	18	limit 17 to yr="2005 - Current"

Table S2.2 Full search strategy for EMBASE (through Ovid; date of last search – 10/06/2020)

Theme or description		Search terms
Diet	1	exp diet/ OR exp food intake/ OR exp feeding behavior/ OR food/ OR dairy product/ OR dietary fiber/ OR dietary supplement/ OR egg/ OR fast food/ OR fat/ OR fruit/ OR health food/ OR meat/ OR nut/ OR sugar/ OR vegetable/
	2	diet*.ti,ab,kw.
	3	nutriti*.ti,ab,kw.
	4	food.ti,ab,kw.
	5	eat*.ti,ab,kw.
	6	energy intake.ti,ab,kw.
	7	(macronutrient* OR micronutrient* OR nutrient*).ti,ab,kw.
	8	1 OR 2 OR 3 OR 4 OR 5 OR 6 OR 7
Depression	9	mood disorder/ OR depression/
	10	(depression* OR (depressive adj3 (condition* OR disorder* OR symptom*))).ti,ab,kw.
	11	9 OR 10
Type of study	12	systematic review/ OR meta analysis/
	13	(systematic adj3 review).ti,ab,kw
	14	meta-analysis OR (meta ADJ2 analysis).ti,ab,kw
	15	12 OR 13 OR 14
Combining search themes	16	8 AND 11 AND 15
Language	17	limit 16 to (english or french)
Search years	18	limit 17 to yr="2005 - Current"

Supplementary file 2. Search strategy for all databases (umbrella review)

Table S2.3 Full search strategy for PsycINFO (through Ovid; date of last search – 10/06/2020)

Theme or description		Search terms
Diet	1	exp appetite/ OR diets/ OR food intake/ OR eating behavior/
	2	diet*.mp.
	3	nutriti*.mp.
	4	food.mp.
	5	eat*.mp.
	6	energy intake.mp.
	7	(macronutrient* or micronutrient* or nutrient*).mp.
	8	1 OR 2 OR 3 OR 4 OR 5 OR 6 OR 7
Depression	9	major depression/ OR dysthymic disorder/ OR recurrent depression/ OR treatment resistant depression/
	10	(depression* OR (depressive ADJ3 (condition* OR disorder* OR symptom*))).mp.
	11	9 OR 10
Type of study	12	systematic review/ OR meta analysis/
	13	(systematic ADJ3 review).mp.
	14	meta-analysis OR (meta ADJ2 analysis).mp.
	15	12 OR 13 OR 14
Combining search themes	16	8 AND 11 AND 15
Language	17	limit 16 to (english or french)
Search years	18	limit 17 to yr="2005 - Current"

Table S2.4 Full search strategy for Cochrane Database of Systematic Reviews and the Joanna Briggs Institute EBP Database (through Ovid; date of last search – 10/06/2020)

Theme or description		Search terms
Diet	1	diet*.mp.
	2	nutriti*.mp
	3	food.ti,mp.
	4	eat*.mp.
	5	energy intake.mp.
	6	(macronutrient* OR micronutrient* OR nutrient*).mp.
	7	1 OR 2 OR 3 OR 4 OR 5 OR 6
Depression	8	(depression* OR (depressive ADJ3 (condition* OR disorder* OR symptom*))).mp.
Combining search themes	9	7 AND 8
Search years	10	limit 9 to yr="2005 - Current"

Table S2.5 Full search strategy for Web of Science (date of last search – 10/06/2020)

Theme or description		Search terms
Diet	1	TS=(diet* OR nutri* OR food OR eat* OR energy intake OR macronutrient* OR *micronutrient* OR nutrient*)
Depression	2	TS=(depression* OR (depressive NEAR (condition* OR disorder* OR symptom*)))
Type of study	3	TS=(systematic review OR (systematic NEAR review) OR meta-analysis OR (meta NEAR analysis))
Combining search themes	4	#1 AND #2 AND #3
Language	5	#4 AND LANGUAGE: (English OR French)
Search years	6	Timespan=2005-2020
