# Supplementary material for: Gathering the Evidence on Diet and Depression: A Protocol for an Umbrella Review and Updated Meta-Analyses
Source: Methods Protoc. 2023 Aug 31;6(5):78. doi: 10.3390/mps6050078 (PMC10514888; doi:10.3390/mps6050078)
Supplement: Supplementary file 1 [file mps-06-00078-s001.zip › Supplementary file 3. Search strategy for all databases (U).pdf]

Supplementary file 3. Search strategy for all databases (updated meta-analyses)

Table S3.1 Full search strategy for Medline (through Ovid)

Theme or description		Search terms
Dietary pattern	1	exp diet/
	2	(diet OR diet* pattern* OR diet* quality).ti,ab,kw.
	3	eating pattern*.ti,ab,kw.
	4	food pattern*.ti,ab,kw.
	5	1 OR 2 OR 3 OR 4
Depression	6	exp depressive disorder/ OR depression/ OR mood disorders/
	7	(depression* OR (depressive ADJ3 (condition* OR disorder* OR symptom*))).ti,ab,kw.
	8	6 OR 7
Combining search themes	9	5 AND 8
Language	10	limit 9 to (english or french)
Search years	11	limit 10 to yr="2 years prior last SR publication - date of search"

Table S3.2 Full search strategy for EMBASE (through Ovid)

Theme or description		Search terms
Dietary pattern	1	exp diet/
	2	(diet OR diet* pattern* OR diet* quality).ti,ab,kw.
	3	eating pattern*.ti,ab,kw.
	4	food pattern*.ti,ab,kw.
	5	1 OR 2 OR 3 OR 4
Depression	6	mood disorder/ OR depression/
	7	(depression* OR (depressive ADJ3 (condition* OR disorder* OR symptom*))).ti,ab,kw.
	8	6 OR 7
Combining search themes	9	5 AND 8
Language	10	limit 9 to (english or french)
Search years	11	limit 10 to yr="2 years prior last SR publication - date of search"

Table S3.3 Full search strategy for PsycINFO (through Ovid)

Theme or description		Search terms
Dietary pattern	1	diets/
	2	(diet OR diet* pattern* OR diet* quality).mp.
	3	eating pattern*.mp.
	4	food pattern*.mp.
	5	1 OR 2 OR 3 OR 4
Depression	6	major depression/ OR dysthymic disorder/ OR recurrent depression/ OR treatment resistant depression/
	7	(depression* OR (depressive ADJ3 (condition* OR disorder* OR symptom*))).mp.
	8	8 OR 9
Combining search themes	9	5 AND 8
Language	10	limit 9 to (english or french)
Search years	11	limit 10 to yr="2 years prior last SR publication - date of search"

Table S3.4 Full search strategy for the Cochrane Central Register of Controlled Trials

Theme or description		Search terms
Dietary pattern	1	(diet OR diet* pattern* OR diet* quality).mp.
	2	eating pattern*.mp.
	3	food pattern*.mp
	4	1 OR 2 OR 3
Depression	5	(depression* OR (depressive ADJ3 (condition* OR disorder* OR symptom*))).mp.
Combining search themes	6	4 AND 5
Search years	7	limit 6 to yr="2 years prior last SR publication - date of search"

Supplementary file 3. Search strategy for all databases (updated meta-analyses)

Table S3.5 Full search strategy for Web of Science

Theme or description		Search terms
Dietary pattern	1	TS=(diet OR diet* pattern* OR diet* quality OR eating pattern* OR food pattern*)
Depression	2	TS=(depression* OR (depressive ADJ3 (condition* OR disorder* OR symptom*)))
Combining search themes	3	#1 AND #2
Language	4	#3 AND LANGUAGE: (English OR French)
Search years	5	Timespan=2 years prior last SR publication - date of search
